# Clinical utility of HCV core antigen detection and quantification using serum samples and dried blood spots in people who inject drugs in Dar-es-Salaam, Tanzania

**DOI:** 10.7448/IAS.20.1.21856

**Published:** 2017-09-19

**Authors:** Zameer Mohamed, Jessie Mbwambo, Yusuke Shimakawa, Lila Poiteau, Stéphane Chevaliez, Jean-Michel Pawlotsky, John Rwegasha, Sanjay Bhagani, Simon D Taylor-Robinson, Julie Makani, Mark R Thursz, Maud Lemoine

**Affiliations:** ^a^ Department of Hepatology, Imperial College London, St Mary’s Hospital, London, UK; ^b^ Department of Psychiatry, Muhimbili University of Health and Allied Sciences, Muhimbili National Hospital, Dar es Salaam, Tanzania; ^c^ Unité d’Épidémiologie des Maladies Émergentes, Institut Pasteur, Paris, France; ^d^ French National Reference Center for Viral Hepatitis B, C and delta; Department of Virology; Hopital Henri Mondor, Université Paris-Est, Créteil, France; ^e^ Department of Gastroenterology, Muhimbili National Hospital, Dar es Salaam, Tanzania; ^f^ Department of HIV Medicine and Infectious Diseases, The Royal Free Hospital, London, UK; ^g^ Department of Haematology, Muhimbili University of Health and Allied Sciences, Muhimbili National Hospital, Dar es Salaam, Tanzania

**Keywords:** people who inject drugs, hepatitis C virus (HCV), dried blood spot, HCV core antigen, screening, Africa

## Abstract

**Introduction**: A lack of access to hepatitis C virus (HCV) diagnostics is a significant barrier to achieving the World Health Organization 2030 global elimination goal. HCV core antigen (HCVcAg) quantification and dried blood spot (DBS) are appealing alternatives to conventional HCV serology and nucleic acid testing (NAT) for resource-constraint settings, particularly in difficult-to-reach populations. We assessed the accuracy of serum and DBS HCVcAg testing in people who inject drugs in Tanzania using HCV NAT as a reference.

**Method**: Between May and July 2015, consecutive HCV-seropositive patients enrolled in the local opioid substitution treatment centre were invited to participate in the study. All had HCV RNA detection (Roche Molecular Systems, Pleasanton, CA, USA), genotyping (NS5B gene phylogenetic analysis) and HCVcAg on blood samples and DBS (Architect assay; Abbott Diagnostics, Chicago, IL, USA).

**Results**: Out of 153 HCV-seropositive individuals, 65 (42.5%) and 15 (9.8%) were co-infected with HIV (41 (63%) were on anti-retroviral therapy (ARVs)) and hepatitis B respectively. In total, 116 were viraemic, median viral load of 5.7 (Interquartile range (IQR); 4.0–6.3) log iU/ml (75 (68.2%) were genotype 1a, 35 (31.8%) genotype 4a). The median alanine transaminase (ALT) (iU/l), aspartate transaminase (AST) (iU/l) and gamma-glutamyl transferase (GGT) (iU/l) were 35 (IQR; 23–51), 46 (32–57) and 69 (35–151) respectively. For the quantification of HCV RNA, serum HCVcAg had a sensitivity at 99.1% and a specificity at 94.1%, with an area under the receiver operating curve (AUROC) at 0.99 (95% CI 0.98–1.00). DBS HCVcAg had a sensitivity of 76.1% and a specificity of 97.3%, with an AUROC of 0.87 (95% CI 0.83–0.92). HCVcAg performance did not differ by HIV co-infection or HCV genotype.

**Conclusions**: Our study suggests that HCVcAg testing in serum is an excellent alternative to HCV polymerase chain reaction in Africa. Although HCVcAg detection and quantification in DBS has a reduced sensitivity, its specificity and accuracy are good and it could therefore be used for scaling up HCV testing and care in resource-limited African settings.

## Introduction

An estimated 80 million people are chronically infected with hepatitis C virus (HCV) worldwide and about 700,000 annual deaths are attributable to HCV [1–3]. The World Health Organization (WHO) has recently called for HCV elimination and aimed for a reduction in incidence by 90% and HCV-related mortality by 65% by 2030 [4]. As part of this manifesto the WHO has called for scaling up interventions to improve HCV screening and linkage-to-care of high-risk populations, including people who inject drugs (PWIDs) [5].

Achieving these objectives will be extremely challenging in low-and-middle-income countries (LMICs) where less than 5% of people are aware of their HCV status and access to diagnostic tests is very limited [6].

Traditionally diagnosis of HCV infection relies on a two-step process constituting serology (enzyme immunoassay (EIA)), followed by a nucleic acid test (NAT) to confirm the infection and quantify the viral load. However, 40% of LMICs report no access to NAT which is expensive and requires high-quality laboratories and well-trained staff [5,7].

HCV core antigen (HCVcAg) quantification using an inexpensive commercial chemiluminescent microparticle assay (CMA) has been shown to be a reliable surrogate for HCV RNA measurement [8] and therefore might be a good alternative to HCV RNA measurement in resource-limited countries [9,10].

Decentralization of testing and care is critical to scale up HCV screening and treatment. The use of dried blood spot (DBS) has proven an important asset in the provision of HIV care in Africa and a recent review emphasized the value of HCV DBS testing in high-risk populations. DBS has the added benefit of being able to be obtained from a finger-prick, overcoming any challenges of difficult venous access in PWIDs [11].

In sub-Saharan African (SSA) the use of injectable drugs has been long considered as a minor issue. However, injecting drug use has been recognized as a growing concern in coastal East Africa, in particular Kenya and Tanzania where the prevalence of HCV in PWIDs might exceeds 40% [12–14]. HCVcAg testing using DBS might be an interesting screening method to scale up HCV screening in Africa, in particular in difficult-to-reach populations such as PWIDs. However, the accuracy of HCVcAg detection and quantification for HCV viral load measurement has been poorly assessed in Africa and no data on its performance using DBS in Africa are currently available [15].

As part of a screening intervention for HCV infection in PWIDs in Tanzania, Dar-es-Salaam, we assessed the performance of HCVcAg detection and quantification in serum and DBS using conventional HCV RNA NAT as a reference.

## Methods

### Study population

Between February 2011 and June 2015 routine blood borne virus screening was offered to all PWIDs registering at the Methadone clinic at Muhimbili National Hospital (MNH) in Dar-es-Salaam. Between April and July 2015 all those with known positive HCV serology were invited to participate in further virological and clinical evaluation. We collected history of hepatitis B virus (HBV) and HIV infection (including most recent CD4 cell count) from the screening database and obtained blood samples for further virological investigations.

### Ethical consideration

The study received clearance from both Muhumibili University for Health and Allied Sciences and the Tanzanian National Institute for Medical Research (NIMR) (NIMR/HQ/R8a/Vol.ix/2298) institutional review board panels. Participants were enrolled in the study after providing written consent. The study was performed in accordance with the Helsinki declaration.

### Collection of serum and DBS

Immediately after the phlebotomy, whole blood was applied to DBS filter paper (Whatmann 903; GE Healthcare Europe, Freiburg, Germany). DBS samples were left to be dried on a horizontal surface for 1 h and placed in an individual sealed plastic bag. All serum and DBS samples were stored at −80°C on the same day of collection. All samples were transferred, under temperature regulation, to the virology unit at the Henri Mondor Hospital, Paris, France.

### Serological methods used to detect anti-HCV, anti-HIV Abs and HBsAg

Serology testing for anti-HCV, anti-HIV Abs and HBsAg were performed using the Abbott AXSYM system (Abbott Diagnostics, Chicago, IL, USA) according to the manufacturer’s instructions, at the MNH clinical chemistry department, Dar-es-Salaam.

### HCV RNA detection and quantification

HCV RNA was quantified using the Cobas Ampliprep/Cobas TaqMan HCV version 2 (CAP/CTM; Roche Molecular Systems Pleasanton, CA, USA), real-time polymerase chain reaction (PCR) assay. HCV RNA was extracted from 650 µl of serum by means of Cobas Ampliprep automated extractor and the Cobas TaqMan 96 analyser was used to perform the PCR amplification and detection according to the manufacturer’s instructions.

DBS samples underwent extraction after elution into 1–1.5 ml of lysis buffer (Cobas Ampliprep/Cobas TaqMan Specimen Pre-Extraction (SPEX)) at 56°C with gentle agitation for 30 min and centrifuges at 220×*g* for 1 min before use. Also, 650 µl of pre-extraction supernatant was used to perform the CAP/CTM HCV 2.0 assay.

### HCV core antigen and quantification

HCVcAg detection and quantification assays were performed on both DBS and serum samples using a fully automated CMA (Architect HCV antigen assay; Abbott Diagnostics, Chicago, IL, USA) according to the manufacturer’s instructions.

### HCV genotyping

The genotype and subtype were determined by means of the reference method, that is, sequencing of the non-structural 5B region of the HCV genome followed by phylogenetic analysis, as previously described [16].

## Statistical analysis

Characteristics of the study participants were presented by median and interquartile range for the continuous variables and percentage for the categorical variables. The diagnostic accuracy of the HCVcAg assay to diagnose HCV viraemia (determined by quantitative PCR (qPCR)) was assessed using sensitivity and specificity for each of different type of samples (serum and DBS). Area under the receiver operating curve (AUROC) was compared between the type of samples (serum versus DBS). Correlation between quantified HCVcAg levels and HCV RNA levels was evaluated using Pearson’s correlation coefficient.

## Results

### Study population

During the study timeframe, 153 consecutive PWIDs with positive anti-HCV antibody were recruited, of whom 116 (75.8%; 95% CI 68–82%) were viraemic. Viral quantification was performed using conventional NAT and HCVcAg. 15 PWIDs (9.8%) were co-infected with HBV and 65 (42.5%) were co-infected with HIV with a median CD4 cell count of 553/mm^3^ (IQR 187–769), of whom 41 (63.1%) were on antiretroviral therapy. The median HCV RNA was 5.7 log IU/ml (IQR 4.0–6.3log IU/ml). The only genotypes identified were genotypes 1a (68%) and 4a (31%). The characteristics of the study population are summarized in Table 1.

**Table 1.** Characteristics of study HCV viraemic participants

	Recruited (*n* = 116)
Median age, years (IQR)	38 (35–41)
Male sex (%)	107 (92.2)
Anti-HIV positive, n (%)	51 (43.9)
CD4 count, cells/mm^3^ (IQR)	551 (275–675)
No ARV, n (%)	20 (39.2)
Efavirienz/emtricitabine/tenofovir, *n* (%)	17 (33.3)
Lamivudine/zidovudine/efavirenz, *n* (%)	7 (13.7)
Tenofovir/lamivudine/efavirenz, *n* (%)	7 (13.7)
HBsAg positive, *n* (%)	9 (7.8)
Median ALT, IU/l (IQR)	35 (23–51)
Median AST, IU/l (IQR)	46 (32–57)
Median GGT, IU/l (IQR)	69 (35–151)
Median total bilirubin, µmol/l (IQR)	7 (5–10)
Median platelet count, ×10^9^/l (IQR)	188 (151–252)
Positive HCV RNA, *n* (%)	116 (75.8)
Genotype (*n* = 110)	
1a	75 (68.2)
4a	35 (31.8)
Median HCV RNA (log iU/ml)	5.7 (4.0–6.3)
Median HCV core Ag using serum (log fmol/l)	2.9 (1.0–3.5)
Median HCV core Ag using DBS (log fmol/l)	0.8 (0.5–1.4)

### Performance of HCVcAg assay to detect HCV viraemia

Table 2 shows the diagnostic accuracy of HCVcAg assay in both serum and DBS for HCV RNA quantification by HIV status. Overall, the AUROC (0.99; 95% CI 0.98–1.00), sensitivity (99.1%) and specificity (94.4%) of HCVcAg in serum to identify HCV viraemic individuals were excellent. The AUROC of HCVcAg quantification in DBS was very good but lower (0.87 (95% CI 0.83–0.92), *p* < 0.0001) as compared to serum HCVcAg. To quantify HCV viraemia, HCVcAg assay in DBS had a very high specificity of 97.1%, but a modest sensitivity (76.7%).

**Table 2.** Diagnostic accuracy of (i) HCV core Ag in serum to detect HCV RNA in serum and (ii) HCV core Ag in DBS to detect HCV RNA in serum, by HIV co-infection

Index test	HCV core Ag in serum (cutoff 15 iU/ml)	HCV core Ag in DBS (cutoff 3.0 fmol/l)
Reference test	HCV RNA in serum (cutoff 15 iU/ml)	HCV RNA in serum (cutoff 3.0 fmol/l)
1. All, *n* = 153		
AUROC (95% CI)	0.99 (0.98–1.00)*	0.87 (0.83–0.92)*
Sensitivity (%)	99.1% (114/115)	76.7% (89/116)
Specificity (%)	94.4% (34/36)	97.3% (36/37)
Correctly classified (%)	98.0% (148/151)	81.7% (125/153)
PPV (%)	98.3% (114/116)	98.9% (89/90)
NPV (%)	97.1% (34/35)	57.1% (36/63)
Positive/negative LR	17.8/0.009	28.4/0.2
2. HIV-infected, *n* = 65		
AUROC (95% CI)	0.99 (0.96–1.00)	0.88 (0.80–0.96)
Sensitivity (%)	98.0% (50/51)	82.4% (42/51)
Specificity (%)	84.6% (11/13)	92.9% (13/14)
Correctly classified (%)	95.3% (61/64)	84.6% (55/65)
PPV (%)	96.2% (50/52)	97.7% (42/43)
NPV (%)	91.7% (11/12)	59.1% (13/22)
Positive/negative LR	6.4/0.02	11.5/0.2
3. HIV non-infected, *n* = 84		
AUROC (95% CI)	1.00 (1.00–1.00)	0.87 (0.81–0.92)
Sensitivity (%)	100% (63/63)	73.4% (47/64)
Specificity (%)	100% (20/20)	100% (20/20)
Correctly classified (%)	100% (83/83)	80.0% (67/84)
PPV (%)	100% (63/63)	100% (47/47)
NPV (%)	100% (20/20)	54.1% (20/37)
Positive/negative LR	NA/0.0	NA/0.3

N/A: not applicable; AUROC: area under the receiver operator curve; PPV: positive predictive value; NPV: negative positive value; LR: likehood ratio, * *p* value <0.0001.

The overall performance of HCVcAg using serum or DBS did not differ according to HIV co-infection or HCV genotype (1a or 4a) (Tables 2 and 3).

**Table 3.** Performance of HCVcAg in HCV genotypes 1a and 4a

Index test Reference test	HCV core Ag in serum HCV RNA in serum	HCV core Ag in DBS HCV RNA in serum
Genotype 1a (sensitivity)	100% (75/75)	80.0% (60/75)
Genotype 4a (sensitivity)	100% (34/34)	80.0% (28/35)

All the samples genotyped were positive for HCV RNA; and thus it was not possible to calculate specificity by genotype.

### Correlation between HCVcAg levels and HCV RNA levels

**Figure 1.**
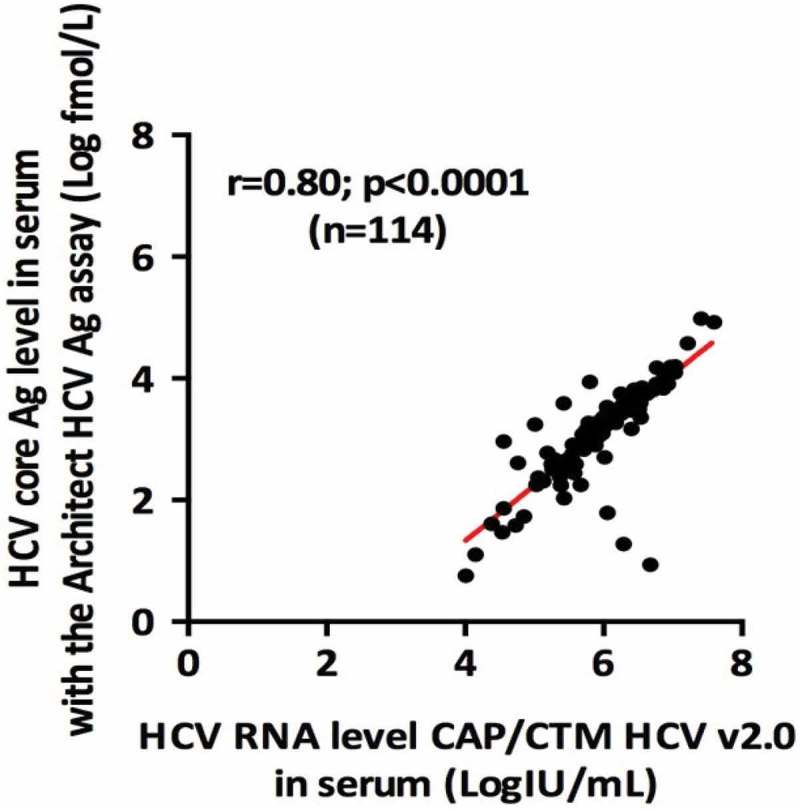
Correlation between serum HCVcAg and serum HCV RNA level.

**Figure 2.**
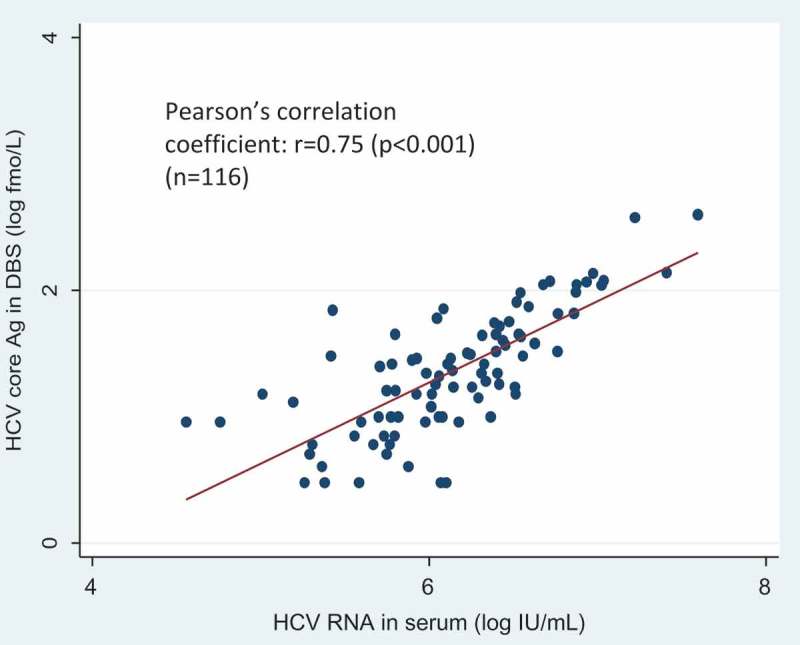
Correlation between HCVcAg in DBS and HCV RNA level.

The correlation between HCVcAg and HCV RNA levels in serum was very strong (*r* = 0.80 *p* < 0.0001, *n* = 114) (Figure 1). When HCVcAg levels were measured on DBS the correlation with HCV RNA in serum remained strong (*r* = 0.75, *p* < 0.001, *n* = 89) (Figure 2).

## Discussion

**Figure 3.**
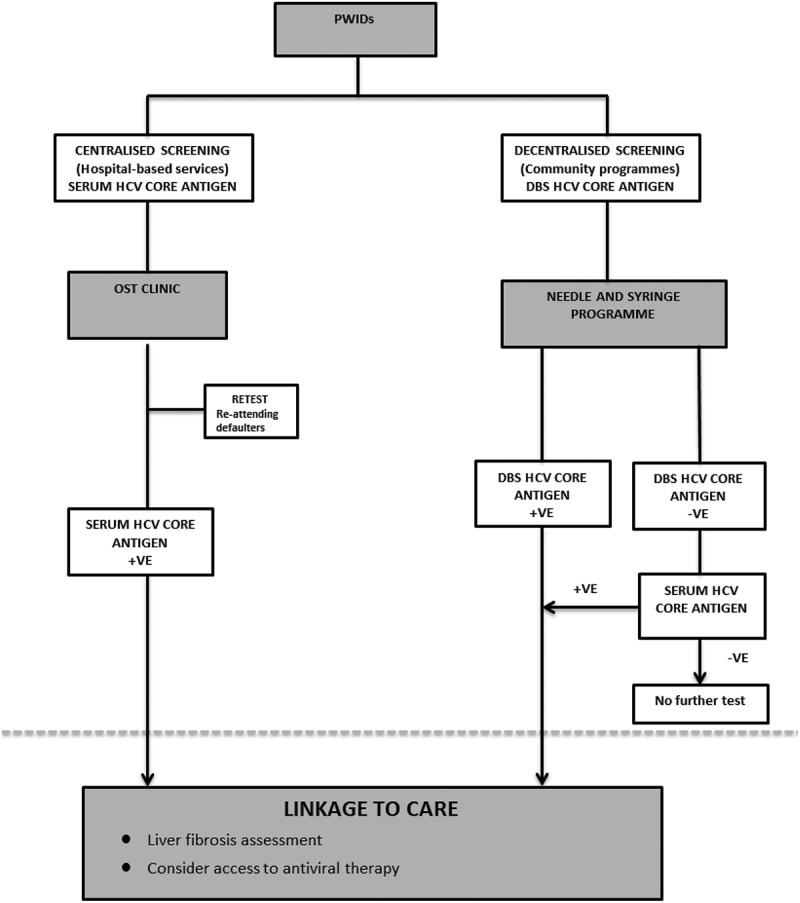
Proposed diagnostic algorithm for screening PWIDs in Africa using serum and DBS HCVcAg.

Among PWIDs positive for anti-HCV antibody in Tanzania, our study reports excellent accuracy of serum HCVcAg assay for HCV RNA detection and quantification irrespective of HCV genotype and HIV co-infection. We also found that the HCVcAg assay performed well using DBS, despite an inferior sensitivity (76.7%) to detect HCV viraemia. This is the first study evaluating the accuracy of HCVcAg using DBS in Africa. Our findings support the development of a simplified HCVcAg-based diagnostic algorithm in PWIDs in SSA (Figure 3).

To date there has been very limited research focused on HCV infection in PWIDs in SSA, with the vast majority of studies focused on HCV seroprevalence [13,17,18]. We found that more than three-quarters (75.8%) of HCV seropositive PWIDs recruited in this study were viraemic, which is within the reported range for SSA (61–77%) [19]. Furthermore, only genotypes 1a and 4a were identified in our study population. This is in line with the sole published study on HCV genotyping in East African PWIDs [14].

The paucity of HCV virological data in SSA is highly related to the limited access to HCV molecular testing. Previous studies performed in high-income countries suggested that HCVcAg might be an alternative to HCV qPCR and called for evaluation in resource-constraint settings [8,20–22],

As previously reported, we found a strong correlation between HCVcAg quantification and HCV RNA levels (*r* = 0.8, *p* < 0.0001) to the manufacturers limit (equivalent to 1,000–3,000 iU/ml) [8,15,21].

Using the Abbott ARCHITECT platform we found excellent performance of serum HCVcAg (AUROC = 0.99, 95% CI 0.98–1.00, sensitivity = 99.1%, specificity = 94.4%) with commercial HCV RNA (Roche) as a reference, irrespective of HCV genotype (1a and 4a). Although we observed a reduction in specificity (84.6%) of serum HCVcAg assay in HIV co-infected patients, the sensitivity did not differ (98% vs. 99.1%) and the AUROC remained excellent (0.99, 95% CI 0.96–1.00).

Using DBS, HCVcAg assay has a significantly lower AUROC but still very good (0.87). Soulier et al. had previously demonstrated a suboptimal performance of HCVcAg in DBS (sensitivity 64.1% and specificity 100%) [21]. In our study the sensitivity of DBS HCVcAg was modest (76.7%), but higher than this reported by Soulier et al. (64%) [21]. In addition, DBS HCVcAg retained a good performance in HIV co-infected samples and the correlation with HCV viral load was sound (*r* = 0.75, *p* < 0.0001) in our study. Thus there is cause for optimism for HCVcAg testing using DBS.

HCVcAg has the benefit of being able to be performed at a lower cost ($10) [23] compared to combined HCV serology and RNA NAT (up to $200) [5], with less sophisticated equipment and skilled staff. However, it still requires a laboratory environment using a dedicated unique machine (Abbott ARCHITECT) [20]. The challenges of performing HCV virological assessment were highlighted by our study, where a lack of access to the appropriate laboratory infrastructure resulted in the shipment of the samples to Europe for processing. Currently, HCVcAg testing can only be carried out at a central location, requiring patients to travel to obtain their diagnosis. This is a fundamental limiter to the scaling up of diagnostic screening in resource-limited settings. DBS testing provides the opportunity to decentralize screening and reach marginalized populations [24]. Drawing parallels from the HIV paradigm, the introduction of DBS testing for HIV viral load proved to be an important contributor to the expansion of HIV treatment in SSA [25]. Thus, the use of DBS should contribute to the expansion of HCV care in resource-limited settings. However to date, only one West African study assessed the validity of HCV EIA and recombinant immunoblot assay using DBS testing in Burkina Faso [26], with no African studies existing evaluating HCV viraemia using DBS.

The current HCV diagnostic algorithm is based on two separate tests (serology and NAT confirmation). The recent WHO guidelines on viral hepatitis screening focused on diagnostic algorithms adapted to resource-constraint settings [5]. Recent work in Cameroon by Duchesne et al. confirmed the practical utility of HCVcAg testing and we agree with their proposed one-step HCVcAg testing in resource-limited settings [15]. In Tanzania there are an estimated 25,000 PWIDs, the majority of whom currently are not engaging in care [13]. Although access to serum HCVcAg assay would be welcome, the use of DBS would overcome the requirement of difficult venepuncture in PWIDs and enable assessment as part of rural needle-and-syringe programmes, maximizing the impact of a HCV screening initiative. Therefore we propose a diagnostic algorithm designed to scale up screening among PWIDs in Africa using HCVcAg serum and DBS testing (Figure 3).

Finally, the validity of HCVcAg in monitoring treatment response to HCV antiviral drugs deserve to be evaluated in PWIDs and more broadly in resource-limited settings. The threshold for viral detection is relatively high in comparison to conventional NAT (3,000 iU/ml vs. 15 iU/ml); however, 95% of viraemic patients with acute or chronic infection would still be correctly identified [27].

Our study has some limitations. Firstly, all samples were collected from the same site and are probably not representative of the whole population of PWIDs in Tanzania. Secondly, due to the small number of HBV co-infected patients, we were unable to perform analysis of HCVcAg performance according to HBV co-infection status. However, there are a number of studies that demonstrated good accuracy in HCV-HBV co-infected subjects [15,28]. Third, the number of HCV-HIV confected was limited (*n* = 65) and the vast majority of patients were maintained on ARVs with high CD4 cells count but unknown levels of HIV viral load. Thus, it will be important to confirm the accuracy of HCVcAg in a larger cohort of HIV/HCV co-infected patients according to HIV RNA levels and the HIV/AIDS CDC stage. Fourth, our study population was infected with only two genotypes (1a and 4a); we therefore cannot confirm the accuracy of HCVcAg assay in different HCV genotypes. Finally, DBS samples in this study were temperature-regulated. A comparison against samples maintained at ambient temperature is warranted, particularly as this is critical to its utility in rural settings.

## Conclusions

Our study suggests that HCVcAg testing in serum is an excellent alternative to HCV PCR in Africa. Although HCVcAg detection and quantification in DBS has a reduced sensitivity, its specificity and accuracy are good and it could therefore be used for scaling up HCV testing and care in resource-limited African settings. Although HCVcAg requires a laboratory equipped with CMA technology, the use of DBS may facilitate the decentralization of HCV testing in Africa, especially in difficult to reach populations.
